# Age‐specific prevalence and genotype distribution of human papillomavirus in women from Northwest China

**DOI:** 10.1002/cam4.4732

**Published:** 2022-04-01

**Authors:** Xiaohong Lin, Liu Chen, Yunyun Zheng, Feng Yan, Jia Li, Jianfang Zhang, Hong Yang

**Affiliations:** ^1^ Department of Gynecology and Obstetrics Xijing Hospital Air Force Medical University (Fourth Military Medical University) Xi'an Shaanxi China

**Keywords:** age‐specific prevalence, genotype distribution, human papillomavirus, Northwest China, women

## Abstract

**Background:**

Human papillomavirus (HPV) is the leading cause of cervical cancer with more than 200 genotypes. Different genotypes have different potentials in causing premalignant lesions and cervical cancers. In this study, we investigated the age‐specific prevalence and genotype distribution of HPV genotypes in Northwest China.

**Materials and Methods:**

We recruited 145,918 unvaccinated women from Northwest China for population‐based HPV DNA screening test during June 2015 to December 2020. And a lab‐based test was performed for each volunteer by flow fluorescent technology to identify the genotypes of HPV.

**Results:**

The overall infection rate of HPV was 22.97%. With the participants divided into 12 groups according to age, a bimodal curve of infection rate was obtained. And the two peaks appeared in the younger than 20 group and 61–65 group, respectively. The five most common HPV genotypes included HPV 16, 58, 52, 53 and 61 in all participants, which were in descending order of frequency. Among women younger than 25 years old, HPV 6 and 11 were more common and even higher than some genotypes mentioned above. Among women older than 65 years old, HPV 18 and 66 were more common than or as high as the six most common genotypes in all populations. Additionally, the distribution of single and multiple infections in each age group was also different.

**Conclusion:**

The baseline prevalence and genotype distribution of HPV in Northwest China was uncovered for the first time. Age was related to the epidemiology of different HPV genotypes. All the results would be of great significance for future healthcare services.

## Graphical abstract

This study provided the baseline prevalence and genotype distribution of HPV in Northwest China for the first time. Age was an influencing factor to the infection rate and prevalence of different HPV genotypes. In addition, the distribution of single and multiple infections also showed age‐related differences.

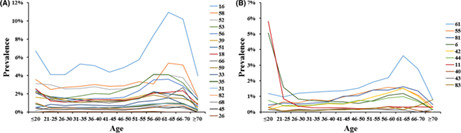

## 1 INTRODUCTION

Human papillomavirus (HPV) is the leading cause of cervical cancer, which is the fourth most common female cancer worldwide.^1^ HPV infection is the most common sexually transmitted infection, and approximately 70% of females having sex will be infected with HPV during the whole lifetime.^2^ Although most HPV infections are asymptomatic, the persistent infection could induce cervical cancer.^3^, ^4^ Hitherto, there are more than 200 HPV genotypes, which are different in respect of the potential to cause premalignant lesions and cervical cancers. Among them, the 12 genotypes, including HPV 16, 18, 31, 33, 35, 39, 45, 51, 52, 56, 58, and 59, were classified as high‐risk (HR) genotypes, and other 12 genotypes, including HPV 6, 11, 40, 42, 43, 44, 54, 61, 70, 72, 81, and CP6108, as low‐risk (LR) genotypes.^5^, ^6^ Specifying the prevalence of different HPV genotypes could predict the cancer risk in the population.

It seems to be a promising method to eliminate cervical cancer by preventing HPV infections among women. Since the development of the first HPV vaccine,^7^ vaccination programs have been spread among women in some developed countries before they get exposed to HPV.^8^, ^9^, ^10^, ^11^ To date, three commercial HPV vaccines are available in China, including the bivalent vaccine (Cervarix) targeting HPV 16 and 18; the quadrivalent HPV vaccine (Gardasil) targeting HPV 6, 11, 16, and 18; and the 9‐valent HPV vaccine (Gardasil) targeting HPV 6, 11, 16, 18, 31, 33, 45, 52, and 58. Furthermore, more HPV vaccines developed by Chinese domestic enterprises are coming to the market.^12^ With the wide variety of vaccines, it is difficult for the public to choose. The prevalence of HPV genotypes is dependent on the geographic region,^13^ so that knowledge of the geographical prevalence of HPV genotypes would provide important information for vaccine selection.

The geographical prevalence of HPV genotypes had been widely investigated in previous studies, leading to different prevalence patterns in different areas. Globally, HPV 16 and 18 are most prevalent.^14^ Additionally, the most common HPV genotypes among the Asian population with cervical cancer are HPV 16, 18, 45, 52, and 58.^15^ However, according to the data from WHO, HPV 16, 18, 33, 52, and 58 are the five most common HPV genotypes in patients with cervical cancer in Eastern Asia.^16^ Data from several provinces in China, such as Guangdong, Jiangsu, Sichuan, Yunnan, Hunan, and Shandong, suggest that the HPV genotypes with high prevalence in different provinces are different.^17^, ^18^, ^19^, ^20^, ^21^ Up to now, studies on HPV genotypes are all conducted in Southwest, Central South, Southeast, or Eastern China, not in Northwest China. In Northwest China, the less‐developed area, the epidemiology of HPV is considered to be different from that of the developed areas. The cost‐effectiveness in the prevention of cervical cancer is of much more attention in Northwest China.^22^ The study for prevalence and genotype distribution of different HPV genotypes is urgent for controlling the economic burden of cervical cancer on public health in Northwest China.

Therefore, we aimed to provide large‐scale epidemiologic data on genotype distribution and prevalence of HPV among women in Northwest China. In total, all samples of this study were collected from women volunteers who had never been vaccinated with HPV vaccines. Hence, the prevalence and distribution of HPV genotypes in Northwest China had been elucidated for the first time, and the age‐related differences were uncovered.

## 2 SUBJECTS AND METHODS

### 2.1 Subjects

Women volunteers who attended Xijing Hospital from June 2015 to December 2020 were enrolled. Inclusion criteria: (1) women who had an intact cervix; (2) women who did not receive cauterization or surgery; (3) women without pregnancy. Exclusion criteria: (1) women who had previous diagnosis or treatment for the cervical or vaginal disease; (2) women who had been vaccinated with any HPV vaccines before. Finally, there were 145,918 women for HPV genotypes DNA screening test. All subjects freely signed informed consent. We have obtained approval from the Ethical Research Committee of Xijing Hospital.

### 2.2 HPV detection and genotyping

Exfoliated endocervical cells were obtained by pathologists with a cervical sampling brush (Jiangsu Jiangyou Medical Science and Technology Corporation). DNA extraction, PCR amplification, hybridization, and HPV genotyping were all conducted with High Throughput HPV genotyping Kits (Tellgen Corporation) by flow fluorescent technology according to the manufacturer's instructions. There were 27 distinguished HPV genotypes in one test. After experiments, 17 HR‐HPV genotypes included HPV 16, 18, 26, 31, 33, 35, 39, 45, 51, 52, 53, 56, 58, 59, 66, 68, and 82.^23^ The other 10 genotypes were defined as LR‐HPV genotypes, including HPV 6, HPV 11, HPV 40, HPV 42, HPV 43, HPV 44, HPV 55, HPV 61, HPV81, and HPV 83.

### 2.3 Statistical analysis

SAS statistical software, version 9.4 (SAS Institute Inc.) was carried out for statistical analyses. *p* < 0.05 was considered as statistical significance. The prevalence of HPV infections among groups (≤20, 21–25, 26–30, 31–35, 36–40, 41–45, 46–50, 51–55, 56–60, 61–65, 66–70 and >70 years old) and overall was calculated and compared using Chi‐square test or Fisher's exact test. Then, the prevalence of 27 HPV genotypes was calculated in the whole population and each age group. The prevalence of the HR‐HPV and LR‐HPV genotypes in each age group were combined. Finally, the prevalence of single, double (infected with two HPV genotypes), triple (infected with three HPV genotypes), and multiple HPV infections (infected with four or more HPV genotypes) were calculated in population and each age group, respectively.

## 3 RESULTS

### 3.1 HPV infection rates and age‐specific prevalence

**FIGURE 1:**
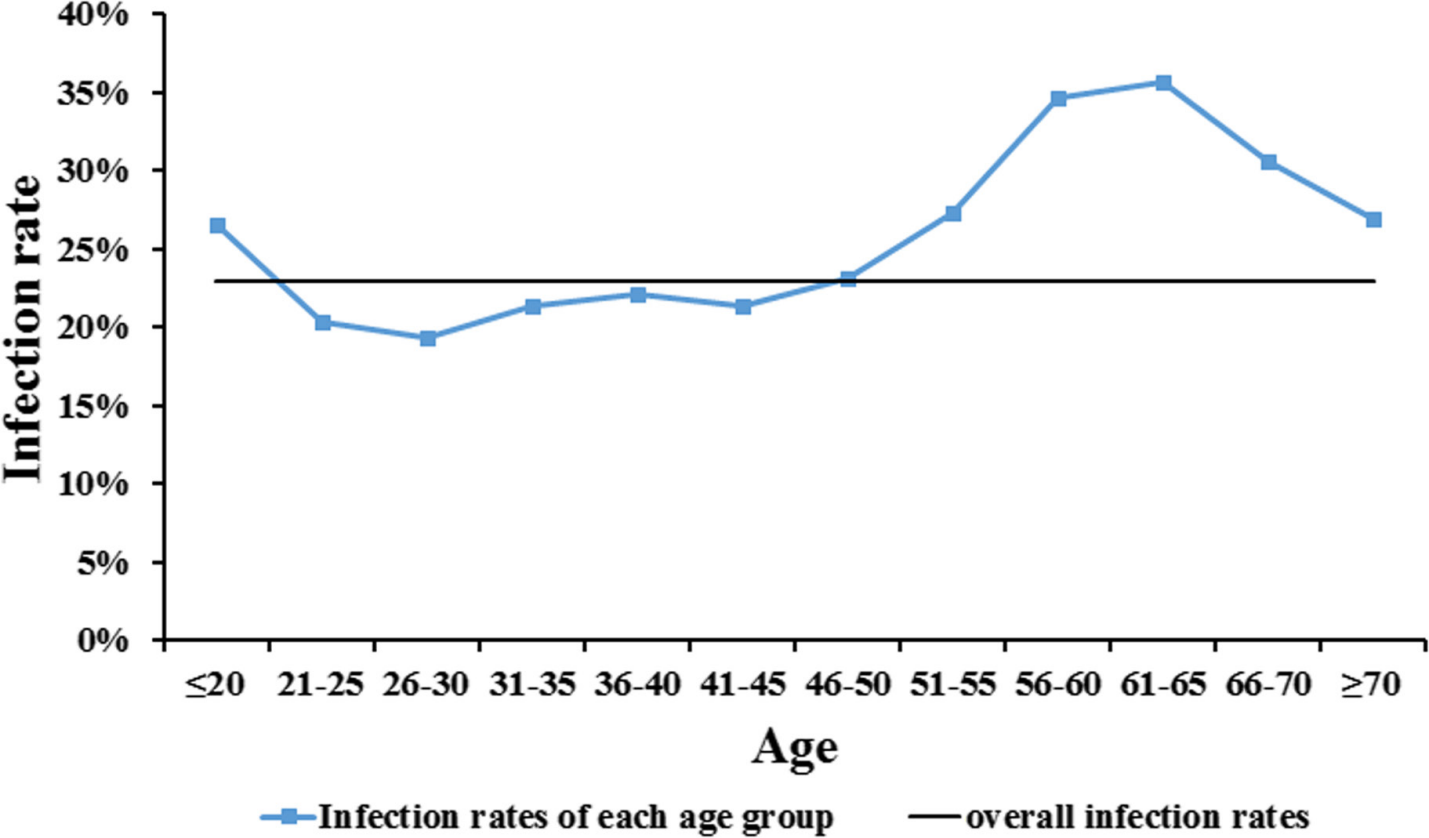
HPV infection rates and age‐specific prevalence

There were 145,918 women (15 to 82 years) included, and 33,522 were HPV positive (Table 1), with an infection rate of 22.97%. All subjects were divided into 12 groups according to age, and the infection rate showed a U‐shape among women younger than 20 to those in 61–65. And among subjects who were 66–70 and older than 70 years old, the infection rate showed a decreasing trend compared with those who were 56–60 and 61–65 years old, but it was higher than the younger (Figure 1). The lowest and highest infection rates were 19.30% and 35.66% in the ages of 26–30 and 61–65 groups, respectively. Among subjects who were between 21 and 45 years old, the infection rate fluctuated between 19.30% and 22.11%, being lower than the overall infection rate; however, the other groups were the opposite.

**TABLE 1:** Basic character of the study population

Character	Number	Percentage
Age (years)		
Median age	40.7 ± 13.3	
Age group (years)		
≤20	672	0.46
21–25	6919	4.74
26–30	19,287	13.22
31–35	22,115	15.16
36–40	22,816	15.64
41–45	26,700	18.30
46–50	22,647	15.52
51–55	13,569	9.30
56–60	6226	4.27
61–65	3141	2.15
66–70	1198	0.82
≥70	628	0.43
HPV‐positive cases		
Overall	33,522	22.97
Infection pattern		
Single infection	25,181	75.38
Double infection	6198	18.22
Triple infection	1593	4.75
Multiple infection (≥4)	550	1.64

Abbreviation: HPV, human papillomavirus.

### 3.2 Genotype distribution of HPV and age‐specific prevalence

The prevalence of 27 genotypes in the population was in details (Supplementary S1). HPV 16 was most prevalent (5.18%, 7564/145,918). After close therewith is HPV 58 (3.10%, 4521/145,918), HPV 52 (2.75%, 4013/145,918), HPV 53 (2.18%, 3181/145,918), and HPV 61 (1.74%, 2532/145,918). Taken together, they were the five most common HPV genotypes in the population. And among the five, only HPV 61 was an LR‐HPV genotype.

**FIGURE 2:**
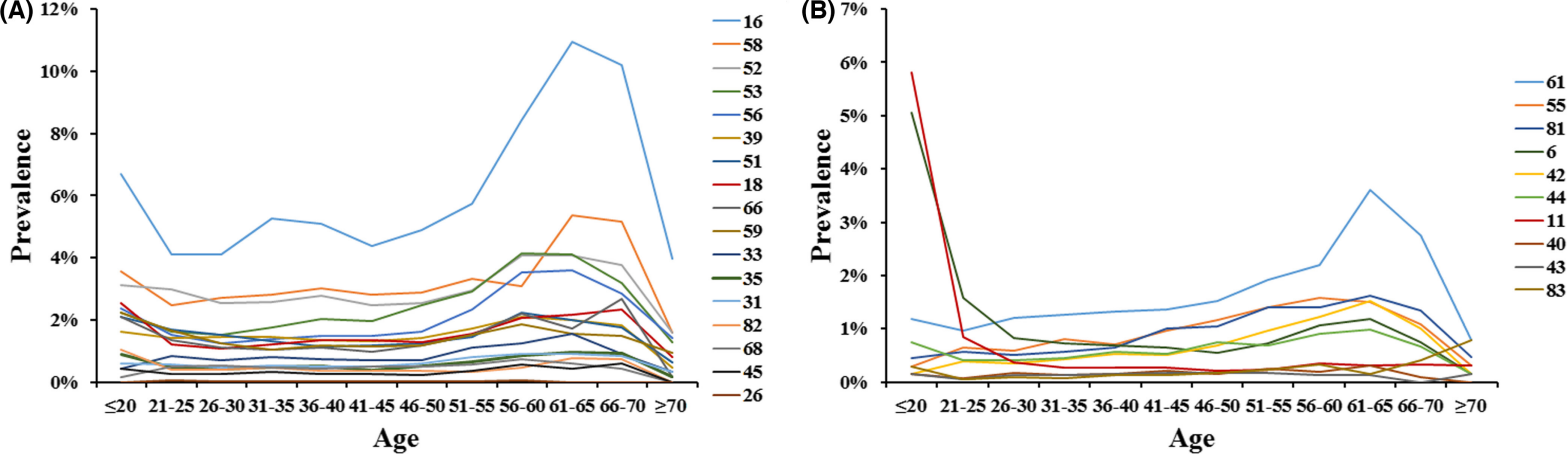
Genotype distribution of HPV in each age group. (A) HR genotypes. (B) LR genotypes. HPV, human papillomavirus; HR, high‐risk; LR, low‐risk

As shown in Figure 2, in women younger than 20, the five most common HR‐HPV genotypes were HPV 16 (6.70%, 45/672), HPV 58 (3.57%, 24/672), HPV 52 (3.13%, 21/672), HPV 18 (2.53%, 17/672), and HPV 56 (2.38%, 16/672). Moreover, HPV 11 (5.80%, 39/672) and HPV 6 (5.06%, 34/672) were the first two LR‐HPV genotypes in this age group. Similarly, in women aged 21–25, HPV 6 and 11 were still the most common LR‐HPV genotypes, and the next one was HPV 61. However, in other age groups, HPV 61 was the most common LR‐HPV genotype. In all HR‐HPV genotypes, HPV 16, 58, and 52 were most prevalent in all age groups. In women older than 36, the five most common HR‐HPV genotypes showed a strong consistency, including HPV 16, 58, 52, 53, and 56. In addition, the three most common LR‐HPV genotypes were HPV 61, 55, and 81 in women older than 36.

**FIGURE 3:**
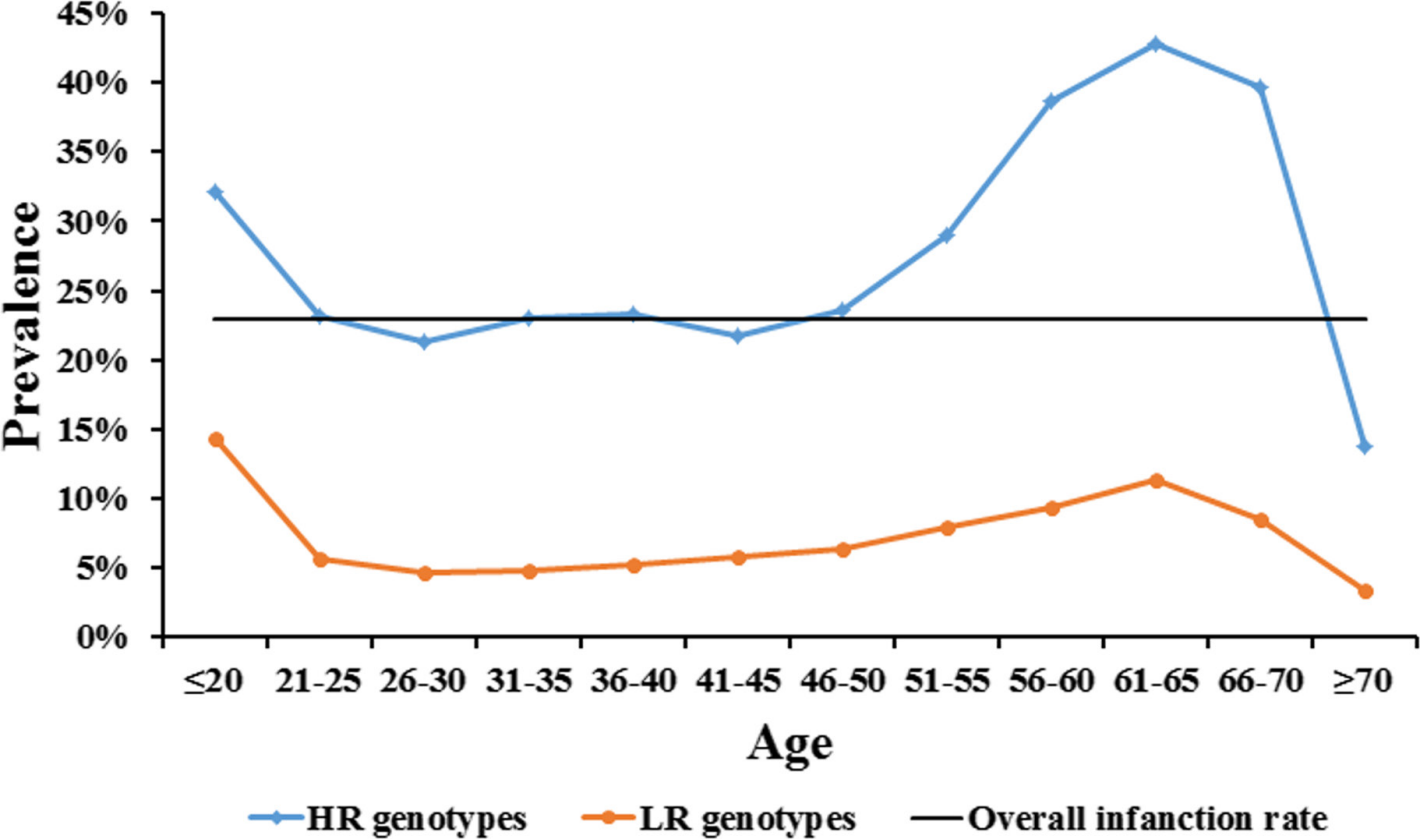
Age‐specific prevalence of HR and LR‐HPV genotypes

The prevalence of HR‐ and LR‐HPV genotypes in different age groups was investigated with combinations of the infections of each HPV genotype. Like age‐specific infection rates, there were U‐shaped curves of both HR‐ and LR‐HPV genotypes (Figure 3), which were significantly decreased in women who were older than 70. In all subjects, the prevalence of LR‐HPV was below the overall infection rate of 20.52%. However, HR‐HPV was more prevalent than the overall infection rate in most subjects, except for the women aged 26–30 and 41–45. And the lowest prevalence of HR‐HPV genotypes was 19.61% in women aged from 26 to 30.

### 3.3 Distribution of single and multiple infections

As some subjects were infected with more than one HPV genotype, the single and multiple infection types were analyzed. A total of 25,181 subjects were single infection with a proportion of 75.38% in all HPV‐positive cases, with its overall prevalence in the population of 17.26% (Table 1). Moreover, the prevalence of double, triple, and multiple infections in the population were 4.25%, 1.09%, and 0.38%, possessing 18.22%, 4.75%, and 1.64% in all HPV‐positive cases, respectively.

**FIGURE 4:**
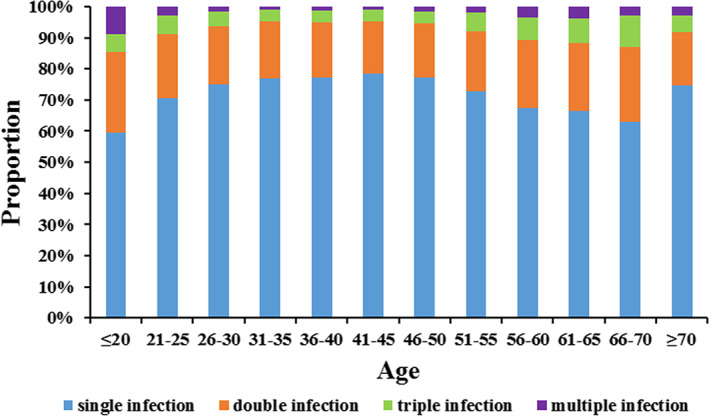
Age‐specific distribution of single and multiple infections

The proportions of single, double, triple, and multiple infections in each age group were further analyzed (Figure 4). The prevalence of multiple infections was also lowest in all age groups except for the women aged younger than 20. And the proportion of multiple infections was 8.94% in this age group, being highest in all age groups. In addition, the proportion of single infection in this age group was 59.22%, being the lowest in all age groups. In other age groups, the proportions of single infection were all above 70%, except for the women aged 56–70. The proportions of double and triple infection were relatively stable except for two peaks that appeared in the women who were younger than 20 and among 56–70. The highest proportions of double and triple infections were 25.70% and 10.11%, appearing in the women who were younger than 20 and among 66–70, respectively.

## 4 DISCUSSION

The HPV distribution and prevalence differ among different populations and regions.^24^, ^25^ As one of the fundamental steps to control cervical cancer, reliable population‐based prevalence, and genotype distributions of HPV are needed for specific areas. Although there are some studies about different regions of China for the prevalence and genotype distribution of HPV, there is still no report in Northwest China.^22^, ^26^, ^27^, ^28^, ^29^, ^30^ Lack of the related statistic data would be not only a great burden for the public health, but also an obstacle for introducing efficient HPV vaccines, as more and more vaccines are available.^12^ In this study, the prevalence of HPV genotypes was uncovered with a large population of 145,918 in Northwest China. And age‐related differences in the prevalence and genotype distribution of HPV were obtained with the large population. All participants had never been vaccinated with any HPV vaccines, and the baseline prevalence of HPV genotypes in this area was obtained for the first time. Our results could not only give a basis for controlling cervical cancer, but also provide a foundation for evaluating the effects of HPV vaccines in the future.

In this study, the overall HPV infection rate was 22.97% in 145,918 women from Northwest China, which was higher compared to the developed regions of China, including 14.7% in Tianjin, 9.9% in Beijing, and 13.3% in Zhejiang.^26^, ^27^, ^31^ Persistent HPV infection is the main cause of cervical cancer, so the higher infection rate of HPV means a higher incidence rate of cervical cancer. Considering that most deaths caused by cervical cancer occur in low‐ and middle‐incoming areas,^32^ cervical cancer could still be an important threat to public health in Northwest China, which is the major less‐developed area of China.

With the population in different age groups, a bimodal curve of infection rate was obtained. The wave trough of the prevalence curve of age‐related HPV appeared in women aged 21 and 45 years, being lower than the overall infection rate. And in women aged older than 51, the infection rate was higher than the younger groups. Although the two peaks may appear in different age groups, the U‐shape curve of the age‐related prevalence of HPV has been observed in many other studies.^33^, ^34^, ^35^ It could be a result of the spontaneous regression of HPV infections in women aged between 21 and 45 years.^36^ We first reported a decreased infection rate in women aged older than 66 after the consistent increase of infection rate in women aged between 46 and 65.

As different HPV genotypes have different carcinogenic potentials, specifying the prevalence of different HPV genotypes is also important for the strategies to prevent and manage cervical cancer.^37^ Although the prevalent genotypes of HPV vary by region worldwide, HPV 16 and 18 are most prevalent with cancer.^15^ As shown by the results, HPV 16 is also the most common HPV genotype in Northwest China.^5^ While, HPV 18 is not prevalent in Northwest China, which could be caused by different sampling standards, as this study is based on population. In a previous study, HPV 18 is not a major prevalent genotype in China.^38^ In contrast, the prevalence of HPV 58 and 52 are just lower than HPV 16 in women from Northwest China in this study, which supports that HPV 16, 58, and 52 are the three most common HPV genotypes in China.^39^ It also suggests that HPV 58 and HPV 52 deserve more attention in Asia.^40^, ^41^ The situation in Northwest China is also unique with a high prevalence of HPV 53 and HPV 61 after the three most common HPV genotypes mentioned above.^17^, ^28^, ^29^, ^30^ Although two LR‐HPV genotypes, HPV 6 and 11, were contained in some vaccines, HPV 61 was the predominant LR‐HPV genotype in Northwest China in this study. All the results provide a basis for developing next ‐generation HPV vaccines.

With the large population included, the relationship between the distribution of HPV genotypes and age was also uncovered. Although the five most prevalent HR‐HPV and three most prevalent LR‐HPV genotypes were all consistent as HPV 16, 58, 52, 53, 56, and HPV 61, 55, 81 in women who were older than 36, the prevalence of some other genotypes were as high as or even higher than these genotypes in younger age groups, such as HPV 39, 51, 18, 6, and 11. Considering the transient infection of HPV in young women with spontaneous cure, it could be concluded that the prevalent HPV genotypes, such as HPV 16, 58, 52, 53, 56, 61, 55, 81, had stronger possibilities of persistent infections in women who were older than 36. In addition, this result also gives guidance for choosing sufficient vaccines for women of different ages. Although HPV 18 is not as prevalent in Chinese women as reported in women from other countries,^42^ its carcinogenicity in Chinese women still needs further study. And a high risk of cervical carcinoma would be expected in women from Northwest China with a higher infection rate of HR‐HPV than LR‐HPV not only in overall, but also in each age group as unraveled by this study. In addition, the single and multiple infection patterns also showed an age‐related difference in this study. All the results suggested that single genotype infection is most common in HPV‐positive cases, which is consistent with former studies.^23^, ^43^ The high peak of single infection appeared in women aged between 21 and 55, which was the trough of the infection rate. These women were also the troughs of double, triple, and multiple infections. Despite the potentially competitive and/or cooperative interactions among different genotypes in coinfections of HPV, it also could be a result of strong immunity in those women. The mechanisms behind the age‐related infection patterns still need further study.

However, there are still some limitations. Previous studies have shown that there are differences in the prevalence and distribution of HPV genotypes between population‐based surveys and cervical carcinoma case investigations in the same area.^18^, ^30^ Therefore, the correlation between HPV genotypes and cervical cytology or histology results in Northwest China needs to be further explored.

## 5 CONCLUSION

In conclusion, the prevalence and distribution of HPV genotypes were investigated in Northwest China for the first time. Age is an influencing factor in the epidemiology of HPV genotypes. Our results provide a basis for future medical intervention. It also provides important information for the development of next‐generation HPV vaccines.

## CONFLICT OF INTEREST

All the authors declare no conflict of interest.

## AUTHOR CONTRIBUTIONS

Xiaohong Lin, Jia Li, Jianfang Zhang, and Hong Yang designed the research. Xiaohong Lin, Liu Chen, and Jianfang Zhang collected the data. Xiaohong Lin, Feng Yan, and Jia Li analyzed the data. Xiaohong Lin, Jia Li, and Jianfang Zhang wrote the manuscript. Hong Yang revised the manuscript into the published version. All authors have read and agreed to the published version of the manuscript.

## Supporting information


Data S1

## Data Availability

The relevant datasets supporting the results of this article are included within the article and its additional files.
